# Invasive liver abscess syndrome caused by *Klebsiella pneumoniae* with definite K2 serotyping in Japan: a case report

**DOI:** 10.1186/s40792-016-0201-2

**Published:** 2016-07-25

**Authors:** Ryota Seo, Daisuke Kudo, Yoshiaki Gu, Hisakazu Yano, Tetsuji Aoyagi, Taku Omura, Shigemi Irino, Mitsuo Kaku, Shigeki Kushimoto

**Affiliations:** 1 Department of Emergency and Critical Care Medicine, Tohoku University Hospital, 1-1 Seiryo-machi, Aoba-ku, Sendai, 980-8574 Japan; 2 Department of Surgery, Yamagata Prefectural Central Hospital, 1800 Aoyagi, Yamagata, 990-2292 Japan; 3 Division of Emergency and Critical Care Medicine, Tohoku University Graduate School of Medicine, 2-1 Seiryo-machi, Aoba-ku, Sendai, 980-8575 Japan; 4 Division of Infection Control and Laboratory Diagnostics, Tohoku University Graduate School of Medicine, 2-1 Seiryo-machi, Aoba-ku, Sendai, 980-8575 Japan; 5 Department of Microbiology and Infectious Diseases, Nara Medical University, 840 Shijo-machi, Kashihara, 634-8521 Japan; 6 Department of Neurology, Tohoku University Graduate School of Medicine, 2-1 Seiryo-machi, Aoba-ku, Sendai, 980-8575 Japan

**Keywords:** *Klebsiella pneumoniae*, Liver abscess, Endogenous endophthalmitis, K2 serotype

## Abstract

**Background:**

*Klebsiella pneumonia* is a well-known human pathogen, and recently, a distinct invasive syndrome caused by *K. pneumoniae* serotypes K1 and K2 has been recognized in Southeast Asia. This syndrome is characterized by primary liver abscess and extrahepatic complications resulting from bacteremic dissemination. We report the first adult case of primary liver abscess caused by the definite K2 serotyped pathogen, with endogenous endophthalmitis in Japan.

**Case presentation:**

A 64-year-old woman was admitted to a nearby hospital for a high fever and diarrhea. She had visual loss of her right eye, renal dysfunction, and thrombocytopenia within 24 h from admission. She was transferred to our institution. On admission, she had no alteration of mental status and normal vital signs; however, she had almost complete ablepsia of the right eye. Laboratory data showed severe inflammation, liver dysfunction, thrombocytopenia, an increased serum creatinine level, and coagulopathy. Computed tomography showed a low density area in the right lobe of the liver. Invasive liver abscess syndrome probably caused by *K. pneumonia* was highly suspected and immediately administered broad-spectrum antibiotics for severe sepsis. Concurrently, endogenous endophthalmitis was diagnosed, and we performed vitrectomy on the day of admission. The blood culture showed *K. pneumoniae* infection. Percutaneous drainage of the liver abscess was also performed. Although she was discharged in a good general condition on day 22, she had complete ablepsia of the right eye. The *K2A* gene was detected by polymerase chain reaction (PCR), which is consistent with the K2 serotype. PCR was also positive for the virulence-associated gene *rmpA*. Final diagnosis was invasive liver abscess syndrome caused by K2 serotype *K. pneumonia*.

**Conclusions:**

Although the primary liver abscess caused by *K. pneumoniae* with a hypermucoviscous phenotype is infrequently reported outside Southeast Asia, physicians should recognize this syndrome, and appropriate diagnosis and treatment is essential for saving patients’ lives and preserving organ function, especially for visual acuity.

## Background

*Klebsiella pneumoniae* is a well-known human pathogen, and recently, a distinct invasive syndrome caused by *K. pneumoniae* serotypes K1 and K2 has been recognized in Southeast Asia, which is becoming an emerging disease worldwide [1].

The invasive nature of some *K. pneumoniae* strains include a hypermucoviscous phenotype associated with serotypes K1 and K2, and the regulator of the mucoid phenotype A gene (*rmpA*) [2]. Of those, *K. pneumoniae* strains expressing the capsular type K1 or K2 antigen have been reported to be especially virulent [3]. These serotypes have a high prevalence of resistance to phagocytosis, intracellular death by neutrophils, and bactericidal complements in a patient’s serum [3].

This invasive syndrome has been reported in Southeast Asian countries [1]. However, only two adult cases caused by *K. pneumoniae* with definite serotyping of K1 [4, 5] and one pediatric case with the K2 serotype [6] have been reported in Japan.

Herein, we present the first adult case with invasive liver abscess syndrome and severe sepsis caused by *K. pneumoniae* with definite K2 serotyping, complicated with endogenous endophthalmitis resulting in complete ablepsia in a healthy woman in Japan.

## Case presentation

**Fig. 1:**
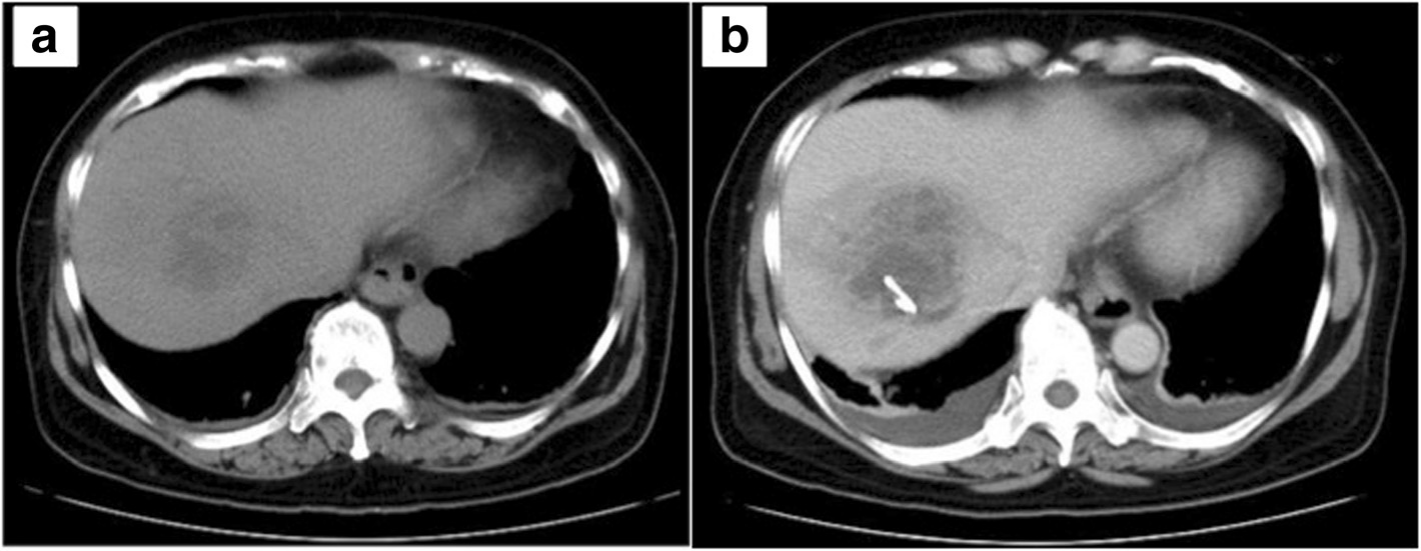
Computed tomography scans. Plain computed tomography showing a low-density lesion in the right lobe of the liver at admission to our hospital (**a**). Enhanced computed tomography showing an unresolved liver abscess on day 6, which was drained percutaneously (**b**)

**Fig. 2:**
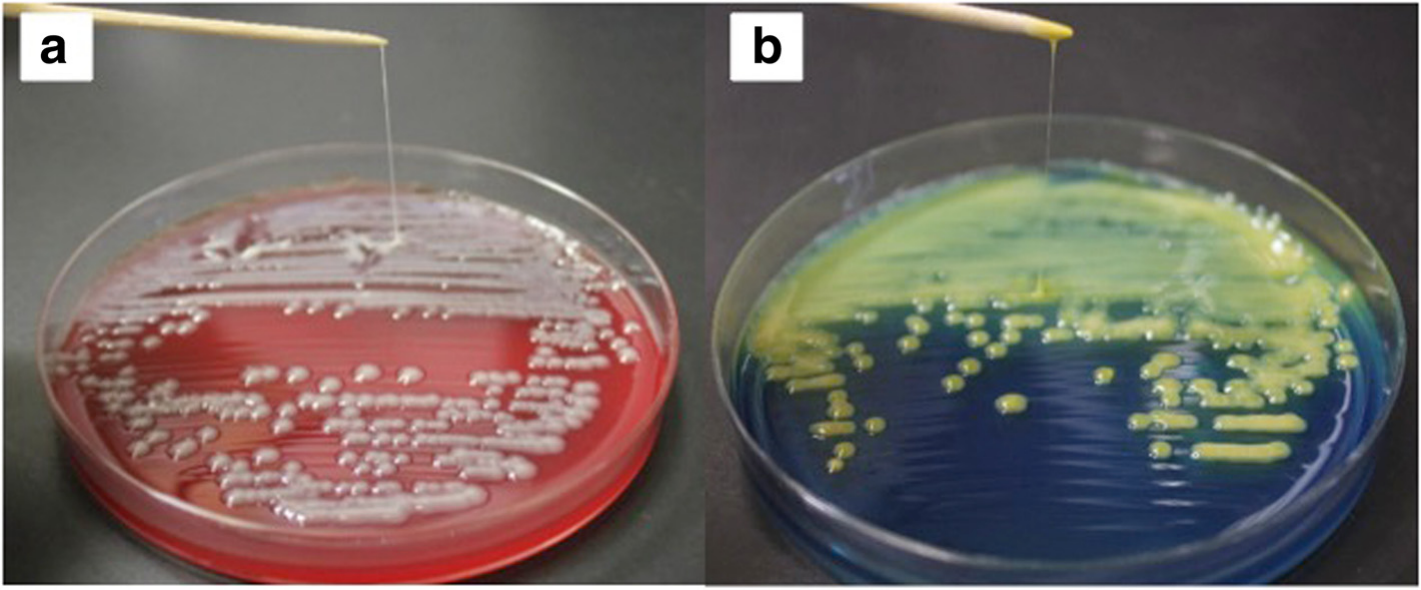
String test. Stretching colonies form a string of >5 mm in length in sheep blood agar (**a**) and in bromothymol blue lactose agar (**b**)

A 64-year-old woman with an unremarkable medical history was admitted to a nearby hospital for a high fever and diarrhea that had lasted for 1 week. She had vision loss in her right eye, exacerbation of renal dysfunction (serum creatinine level, 4.96 mg/dL), and thrombocytopenia (platelet count, 14,000/μL) within 48 h from initial presentation. Therefore, she was transferred to our institution for treatment of severe infectious disease. On admission, she was alert and had no alteration of mental status, no hypoxia on room air, and normal vital signs (temperature, 36.7 °C; blood pressure, 126/80 mmHg; pulse rate, 74 beats/min; respiratory rate, 12 breaths/min). However, she had almost complete ablepsia of the right eye. Laboratory data showed severe inflammation (white cell count, 21,700/μL; C-reactive protein level, 28.2 mg/dL; procalcitonin, 46.4 ng/mL), liver dysfunction (aspartate transaminase, 147 IU/L; alanine transaminase, 345 IU/L), thrombocytopenia (platelet count, 32,000/μL), an increased serum creatinine level (1.35 mg/dL), and coagulopathy (fibrin/fibrinogen degradation product, 56.6 μg/mL; D-dimer, 24.2 μg/mL; prothrombin time-international normalized ratio, 1.15)(Table 1). Computed tomography showed a low density area in the right lobe of the liver, and she was diagnosed with a liver abscess (Fig. 1a). Broad-spectrum antibiotic therapy with meropenem (3 g/day) was immediately started. Endogenous endophthalmitis of the right eye was also diagnosed, and emergency vitrectomy was performed on the day of admission. The blood culture at admission showed *K. pneumoniae*, and the antibiotic therapy was changed to ceftriaxone (2 g/day) on day 4 because all of the cephalosporin had high susceptibility (Table 2) and ceftriaxone was known to penetrate to the liver in high concentration, which was continued to day 14. Percutaneous drainage of the liver abscess, which was unresolved by medical treatment, was performed after coagulopathy improved on day 6 (Fig. 1b). The culture of the drainage fluid also showed *K. pneumoniae*. The drainage tube was removed on day 14, and the patient was discharged in a good general condition on day 22, with an additional 2-week course of oral levofloxacin on days 14–28. However, she had complete ablepsia of the right eye. Capsular probing via PCR for the presence of *magA* (serotype K1) and *K2A* (serotype K2) genes was performed by using the *magA*-specific primers (forward, 5′-GGTGCTCTTTACATCATTGC-3′; and reverse, 5′-GCAATGGCCATTTGCGTTAG-3′) and *k2A*-specific primers (forward, 5′-CAACCATGGTGGTCGATTAG-3′; and reverse, 5′-TGGTAGCCATATCCCTTTGG-3′) as previously described[7]. The virulence-associated gene *rmpA* was also screened using PCR by using the *rmpA*-specific primers (forward, 5′- ACTGGGCTACCTCTGCTTCA-3′; and reverse, 5′- CTTGCATGAGCCATCTTTCA-3′) as previously described [8]. The reaction mixtures of these samples were kept at 95 °C for 5 min, followed by 35 cycles of 95 °C for 1 min, 50 °C for 1 min, and 72 °C for 1 min and then 72 °C for 7 min. The *K2A* gene, which is consistent with the K2 serotype, was detected by PCR. PCR was also positive for the virulence-associated gene *rmpA*. To assess for the presence of hypermucoviscosity, a string test was performed on the organism grown in 5 % sheep blood agar. The formation of a mucous string of >5 mm in length after touching a colony with a loop was considered positive [9]. The isolated *K. pneumoniae* had a positive string test consistent with a hypermucoviscous phenotype (Fig. 2).

**Table 1:** Changes in the patient’s laboratory data

	The previous hospital visit	At admission to our hospital	Day 3	Day 6	Reference ranges
WBC count, /μL	22,900	21,700	14,100	13,400	4,000–9000
CRP, mg/dL	30.3	28.2	7.2	8.6	0.0–0.3
PCT, ng/mL		46.4	5.98	0.88	0.00–0.40
T-Bil, mg/dL	2.19	2	0.9	0.7	0.2–1.0
AST, U/L	206	147	24	25	8–38
ALT, U/L	423	345	96	62	4–43
BUN, mg/dL	57.1	49	13	14	8–20
Cr, mg/dL	1.73	1.35	0.66	0.55	0.44–1.15
PLT count, ×10^4^/μL	1.4	3.2	3.5	16.1	15–35
PT-INR		1.2	1.08	1.15	<1.15
APTT, s		34.5	28.8	30.8	29.6–40.8
FDP, μg/mL		59.2	12.2	9.2	0.0–4.9
D-dimer, μg/mL		24.2	4.7	4.1	0.0–0.9

*WBC* white blood cell, *CRP* C-reactive protein, *PCT* procalcitonin, *T-Bil* total bilirubin, *AST* aspartate transaminase, *ALT* alanine transaminase, *BUN* blood urea nitrogen, *Cr* creatinine, *PLT* platelet, PT-INR, prothrombin time-international normalized ratio, *APTT* activated partial thromboplastin time, *FDP* fibrin/fibrinogen degradation product

**Table 2:** Antimicrobial susceptibility of *Klebsiella pneumoniae*

Antimicrobial agent	MIC (mg/L)	MIC interpretation
Ampicillin	16	R
Piperacillin	8	R
Cefazolin	≦4	S
Cefotaxime	≦1	S
Ceftazidime	≦1	S
Imipenem/cilastatin	≦1	S
Gentamicin	≦1	S
Amikacin	≦2	S
Minocycline	≦1	S
Ciprofloxacin	≦0.25	S
Sulfamethoxazole trimethoprim	≦20	S
Cefepime	≦1	S
Levofloxacin	≦0.12	S
Meropenem	≦0.25	S
Amoxicillin/clavulanic acid	≦2	S
Cefmetazole	≦1	S
Aztreonam	≦1	S
Cefpodoxime	≦0.25	S

*MIC* minimum inhibitory concentration, *S* susceptible, *R* resistant

### Discussion

We present the case of invasive liver abscess syndrome with endogenous endophthalmitis caused by the K2 serotype of *K. pneumoniae* extending to complete ablepsia despite improvement of the pathophysiology of severe sepsis. One systematic review reported *K. pneumoniae* had been the most common causative organism (27 %) in endogenous bacterial endophthalmitis. Following other organisms were *Staphylococcus aureus* (10 %), *Pseudomonas aeruginosa* (6 %), *Group B streptococci* (6 %), and *Neisseria meningitidis* (5 %) [10]. Therefore, use of broad-spectrum antibiotics must be considered until identification of causative microorganism.

This case of a primary liver abscess caused by the definite K2 serotyping of *K. pneumoniae* is the first adult case in Japan. This invasive syndrome caused by *K. pneumoniae* serotypes K1 or K2 has been reported mainly in Southeast Asian countries, especially in Taiwan. We found around 50 cases with liver abscess caused by *K. pneumoniae* serotype K2 all over the world [11, 12]. However, in Japan, only three cases—two in elderly men infected with the K1 serotype and one in a 7-year-old child with the K2 serotype—have been reported [4–6], when we searched PubMed and the Igaku Chuo Zasshi databases for papers published between Jun 01, 1970, and Dec 31, 2015, by using combinations of the following keywords: “*Klebsiella pneumoniae*,” “liver abscess,” “K1” or “K2,” except minutes and selected articles about this invasive syndrome published by Japanese authors.

Almost 30 cases of primary liver abscess caused by *K. pneumoniae* with endogenous endophthalmitis have been reported in Japan (all reports in Japanese); we conducted a search of the medical literature published using the Igaku Chuo Zasshi database and “liver abscess,” “endophthalmitis,” and “*Klebsiella pneumoniae*” as search terms. In addition, we could find two more Japanese literatures related to invasive liver abscess syndrome when we searched Igaku Chuo Zasshi database by using following keywords: “Klebsiella pneumoniae,” “liver abscess,” and “rmpA” [13, 14]. The K1 and K2 serotype were suspected in all cases because of the clinical features, but examination of the serotype has never been reported. Therefore, an invasive liver abscess caused by the K1 or K2 serotype of *K. pneumoniae* may not be rare in Japan.

The virulence-associated gene *rmpA* was positive in our case. *rmpA* is not an independent factor contributing to a liver abscess, but it aids in capsule synthesis. One report showed that all *K. pneumoniae* strains that cause liver abscesses and abscesses at other sites are *rmpA*-positive. *rmpA* has been confirmed as a gene that regulates capsular polysaccharide synthesis [15]. Thus, in our case, *rmpA* was probably associated with liver abscess formation.

## Conclusions

Although a primary liver abscess caused by *K. pneumoniae* with a hypermucoviscous phenotype is infrequently reported outside Southeast Asia, physicians should recognize this syndrome, and appropriate diagnosis and treatment is essential for both saving patients’ lives and preserving organ function, especially for visual acuity.

## Consent

Written informed consent was obtained from the patient for publication of this case report and any accompanying images. A copy of the written consent form is available for review by the Editor-in-Chief of this journal.

## Abbreviations

*K. pneumonia*, *Klebsiella pneumonia*; PCR, polymerase chain reaction; rmpA, mucoid phenotype A gene
